# DockQ: A Quality Measure for Protein-Protein Docking Models

**DOI:** 10.1371/journal.pone.0161879

**Published:** 2016-08-25

**Authors:** Sankar Basu, Björn Wallner

**Affiliations:** 1 Bioinformatics Division, Department of Physics, Chemistry and Biology, Linköping University, Linköping, Sweden; 2 Swedish e-Science Research Center, Linköping University, Linköping, Sweden; Weizmann Institute of Science, ISRAEL

## Abstract

The *state-of-the-art* to assess the structural quality of docking models is currently based on three related yet independent quality measures: F_nat_, LRMS, and iRMS as proposed and standardized by CAPRI. These quality measures quantify different aspects of the quality of a particular docking model and need to be viewed together to reveal the true quality, e.g. a model with relatively poor LRMS (>10Å) might still qualify as 'acceptable' with a descent F_nat_ (>0.50) and iRMS (<3.0Å). This is also the reason why the so called CAPRI criteria for assessing the quality of docking models is defined by applying various *ad-hoc* cutoffs on these measures to classify a docking model into the four classes: Incorrect, Acceptable, Medium, or High quality. This classification has been useful in CAPRI, but since models are grouped in only four bins it is also rather limiting, making it difficult to rank models, correlate with scoring functions or use it as target function in machine learning algorithms. Here, we present DockQ, a continuous protein-protein docking model quality measure derived by combining F_nat_, LRMS, and iRMS to a single score in the range [0, 1] that can be used to assess the quality of protein docking models. By using DockQ on CAPRI models it is possible to almost completely reproduce the original CAPRI classification into Incorrect, Acceptable, Medium and High quality. An average PPV of 94% at 90% Recall demonstrating that there is no need to apply predefined ad-hoc cutoffs to classify docking models. Since DockQ recapitulates the CAPRI classification almost perfectly, it can be viewed as a higher resolution version of the CAPRI classification, making it possible to estimate model quality in a more quantitative way using Z-scores or sum of top ranked models, which has been so valuable for the CASP community. The possibility to directly correlate a quality measure to a scoring function has been crucial for the development of scoring functions for protein structure prediction, and DockQ should be useful in a similar development in the protein docking field. DockQ is available at http://github.com/bjornwallner/DockQ/

## Introduction

Protein-Protein Interactions (PPI) are involved in almost all biological processes. To understand these processes the structure of the protein complex is essential. Despite significant efforts in traditional structural biology and the structural genomics projects that aim at high-throughput complex structure determination [1], the latest statistics from 3did database [2] show that only 7% of the known protein interactions in humans have an associated experimental complex structure. Thus, there is great need for computational methods that predict new interactions and produce high-resolution structural modeling of PPIs. To evaluate the performance of computational methods the quality of the PPI models produced by these methods need to be assessed by comparing their structural similarity to the experimentally solved native structures (targets). In contrast to the protein structure prediction field, where there are several widely accepted quality measures; e.g., C^α^-RMSD, GDT_TS [3], MaxSub [4], TM-score [5], and S-score [6], the IS-score [7] for assessing protein complex models has not achieved wide adoption by the field and the current *state of the art* evaluation protocol for assessing the quality of docking models is still based on three distinct though related measures, namely F_nat_, LRMS and iRMS as proposed and standardized by the Critical Assessment of PRedicted Interactions (CAPRI) community [8]. To calculate these measures, the interface between the two interacting protein molecules (receptor and ligand) is defined as any pair of heavy atoms from the two molecules within 5Å of each other. F_nat_ is then defined as the fraction of native interfacial contacts preserved in the interface of the predicted complex. LRMS is the Ligand Root Mean Square deviation calculated for the backbone of the shorter chain (ligand) of the model after superposition of the longer chain (receptor) [9]. For the third measure, iRMS, the receptor-ligand interface in the target (native) is redefined at a relatively relaxed atomic contact cutoff of 10Å which is twice the value used to define inter-residue 'interface' contacts in case of F_nat_. The backbone atoms of these 'interface' residues is then superposed on their equivalents in the predicted complex (model) to compute the iRMS [9]. For details with pictorial description of all quality measures, see the original reference [10]. The CAPRI evaluation use different cutoffs on these three measures to assign predicted docking models into the four quality classes: Incorrect (F_nat_ < 0.1 or (LRMS > 10 and iRMS > 4.0)), Acceptable ((F_nat_ ≥ 0.1 and F_nat_ < 0.3) and (LRMS ≤ 10.0 or iRMS ≤ 4.0) or (F_nat_ ≥ 0.3 and LRMS > 5.0 and iRMS > 2.0)), Medium ((F_nat_ ≥ 0.3 and F_nat_ < 0.5) and (LRMS ≤ 5.0 or iRMS ≤ 2.0) or (F_nat_ ≥ 0.5 and LRMS > 1.0 and iRMS > 1.0)), or High (F_nat_ ≥ 0.5 and (LRMS ≤ 1.0 or iRMS ≤ 1.0)) [10]. While this classification has been useful for the purpose of CAPRI, it is not as detailed as the quality measures used in the protein structure prediction field, e.g. TM-score and GDT_TS. It is for instance, difficult to directly correlate the CAPRI classification with any scoring function trying to estimate the accuracy of docking models. Thus, the scoring part of CAPRI [8] and benchmarks of scoring functions for docking [11,10,12,13] only focuses on the ability to select good models according to the CAPRI classification, completely ignoring the potential useful information in the lower ranked models in assessing the ability to estimate the true model quality. Thus, there is a need to design a single robust continuous quality estimate covering all different structural attributes captured individually by the CAPRI measures. In this study we derive such a continuous quality measure, DockQ, for docking models that instead of classifying into different quality groups, combines F_nat_, LRMS, and iRMS to yield a score in the range [0, 1], corresponding to low and high quality, respectively. This new measure can essentially be used to recapitulate the original CAPRI classification, and be used for more detailed analyses of similarity and prediction performance.

Furthermore, the recent growth in using machine learning methods to score models would not have been possible if there would not have been a development of single quality measures, like TM-score, GDT_TS and S-score to serve as target functions. These methods have been successful in CASP for predicting the quality of protein structure models [14,15] and there is no reason to believe that they will not be as successful in predicting the quality of docking models. Although the individual CAPRI measures (F_nat_, LRMS, iRMS) as well as the classification into incorrect, acceptable, medium and high quality models could potentially be used as target functions in regression or classification schemes, it is natural and more convenient to use the combined single measure, DockQ, which covers all the different quality attributes, captured by the individual CAPRI measures. The potential use of DockQ as a target function in the design of a docking scoring function by training support vector regression machines to predict quality of docking models has already been demonstrated in a separate study [16].

## Materials and Methods

### Training set

A set from a recent benchmark of docking scoring function [13], (the *MOAL-set*), was used to design and optimize DockQ. This set contained 56,015 docking models for 118 targets from the protein-protein docking Benchmark 4.0 [17], constructed using SwarmDock [18] graciously provided by the authors of Moal et al [13]. The set contained 54,324 incorrect, 762 acceptable, 855 medium, and 74 high quality models.

### Testing set

For independent testing, a subset based on the CAPRI Score_set [19] (http://cb.iri.univ-lille1.fr/Users/lensink/Score_set/) containing models submitted to CAPRI between 2005–2014 with their respective CAPRI quality measure (F_nat_, LRMS, iRMS) was assembled. For simplicity, two targets with multiple correct chain packings, i.e. same sequence binding at two different locations, were removed (Target37: 2W83, Target 40: 3E8L). The final *CAPRI-set* contained 13,849 incorrect, 632 acceptable, 565 medium, and 282 high quality models, in total 15,328.

### Performance Measures

*Matthews Correlation Coefficient (MCC)* is defined by

$$\text{MCC}=\left(\text{TP}\times \text{TN-FP}\times \text{FN}\right)\text{/}{\left[\left(\text{TP}+\text{FP}\right)\left(\text{TP}+\text{FN}\right)\left(\text{TN}+\text{FP}\right)\left(\text{TN}+\text{FN}\right)\right]}^{\text{1/2}}$$

where TP, FP, TN and FN refer to True Positives, False Positives, True Negatives and False Negatives respectively. MCC is defined in the range of -1 (perfect anti-correlation) to 1 (perfect correlation).

*Precision (PPV)* is the ratio of the true positives predicted at a given cutoff and the total number of test outcome positives (including both true and false positives) determined at the same cutoff. Thus, PPV = TP/(TP+FP).

*Recall (TPR)* is the number of true positives predicted at a given cutoff divided by the total number of positives (P = TP + FN) in the set. Thus, TPR = TP/P.

*F1-score* is the harmonic mean between PPV and TPR and could be interpreted as a trade-off between PPV and TPR and is defined by the following equation: F1 = 2PPV× TPR/(PPV+TPR).

### Transforming LRMS and iRMS

To avoid the problem of arbitrarily large RMS values that are essentially equally bad, RMS values were scaled using the inverse square scaling technique adapted from the S-score formula [6]

$$RMS_{scaled}\left(RMS,d_{i}\right)=\frac{1}{1+{\left(\frac{RMS}{d_{i}}\right)}^{2}}$$ (1)

where *RMS*_*scaled*_ represents the scaled RMS deviations corresponding to any of the two terms, LRMS or iRMS (*RMS*) and *d*_*i*_ is a scaling factor, *d*_*1*_ for LRMS and *d*_*2*_ for iRMS, optimized to d_1_ = 8.5Å and d_2_ = 1.5Å (see Results and Discussion).

The hallmark of inverse-square scaling is the asymptotic smooth declination of the scaled function (Y) with gradual increase of the raw score (X) (Figure A in S1 File). While, conversely, the relative increment (dY/dX) of the scaled-to-the-raw-values increases at the lower end of X, represented by a significantly steeper slope on the higher end of Y (say, Y>0.5). The scaling technique thus makes the scaled RMSD functions considerably more sensitive in discriminating between 'good' models (e.g., acceptable vs. medium; or, medium vs. high) varying slightly in their relative quality. While, on the other hand, the function is close to zero for all kinds of 'bad' (incorrect) models regardless of their relative quality.

## Results and Discussion

The aim of this study was to derive a continuous quality measure that can be used to rank docking models and compare performances of methods scoring docking models in a direct way. To make it simple and promote wide-acceptance, we chose to base the scoring function, named DockQ, on the already established quality measures for docking F_nat_, LRMS, and iRMS used in CAPRI [8] and other benchmarks [13]. In the DockQ score we combined F_nat_, LRMS, and iRMS into one score by the mean of F_nat_, and the two RMS values scaled according to Eq 1.

$$DockQ\left(F_{nat},LRMS,iRMS,d_{1},d_{2}\right)=\left(F_{nat}+RMS_{scaled}\left(LRMS,d_{1}\right)+RMS_{scaled}\left(iRMS,d_{2}\right)\right)/3$$ (2)

where *RMS*_*scaled*_*(RMS*,*d)* is defined in Eq 1, *d*_*1*_ and *d*_*2*_ are scaling parameters that determines how fast large RMS values should be scaled to zero, and needs to be set based on the score range for LRMS and iRMS. The advantages of the non-linear scaling of the RMS values is that the function (Eq 2) only contains terms between 0 and 1, and that all have the same dependence on quality, the higher the better. Perhaps even more important is that RMS values that should be considered equally bad e.g. iRMS of 7Å or 14Å both get essentially the same low RMS_scaled_ score.

### Optimizing d_1_ and d_2_

**Fig 1:**
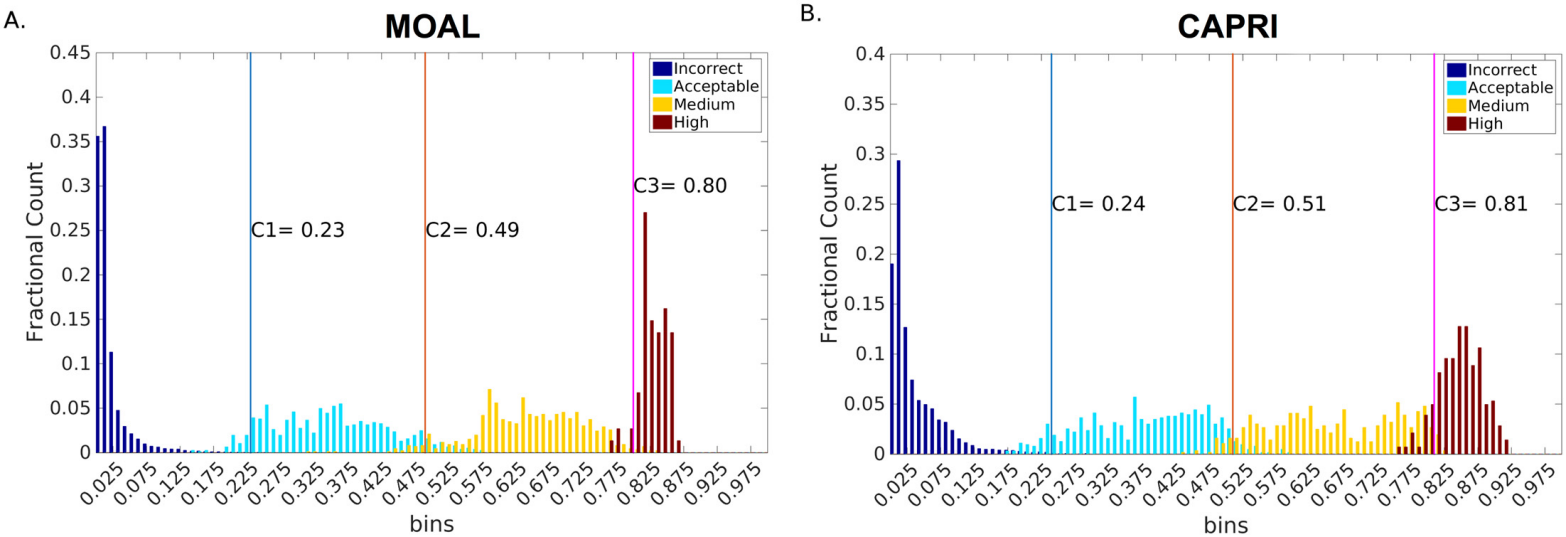
Distribution of DockQ score for Incorrect, Acceptable, Medium, and High quality models, respectively for (A) MOAL-set, and (B) CAPRI-set. The colored bars corresponding to each CAPRI class represents frequency distribution of models predicted to be falling in a particular class normalized by the total number of models in that class.

The two parameters in the DockQ score, *d*_*1*_ and *d*_*2*_, were optimized in a grid search on the MOAL-set by calculating Eq 2 for all pairs of *d*_*1*_ and *d*_*2*_ in the range 0.5 to 10Å for *d*_*1*_, and 0.5 to 5Å for *d*_*2*_ in steps of 0.5. For each *(d*_*1*_,*d*_*2*_*)* pair the ability to separate the models according the CAPRI classification was assessed by first defining the three cutoffs, C1, C2, and C3, that optimized the Matthew's correlation coefficient (MCC) between, Incorrect and Acceptable (C1), Acceptable and Medium (C2), and Medium and High (C3), respectively. The optimized cutoffs were used to calculate an F1-score for the classification performance for each of the four different classes. Finally, the average F1-score was used to measure the overall classification performance and to decide on d_1_ and d_2_. The maximum average F1-score (0.91) was obtained for d_1_ = 8.5Å and d_2_ = 1.5Å (Figure B in S1 File), corresponding to the cutoffs C1 = 0.23, C2 = 0.49, and C3 = 0.80 (Fig 1A).

### Independent Benchmark

**Fig 2:**
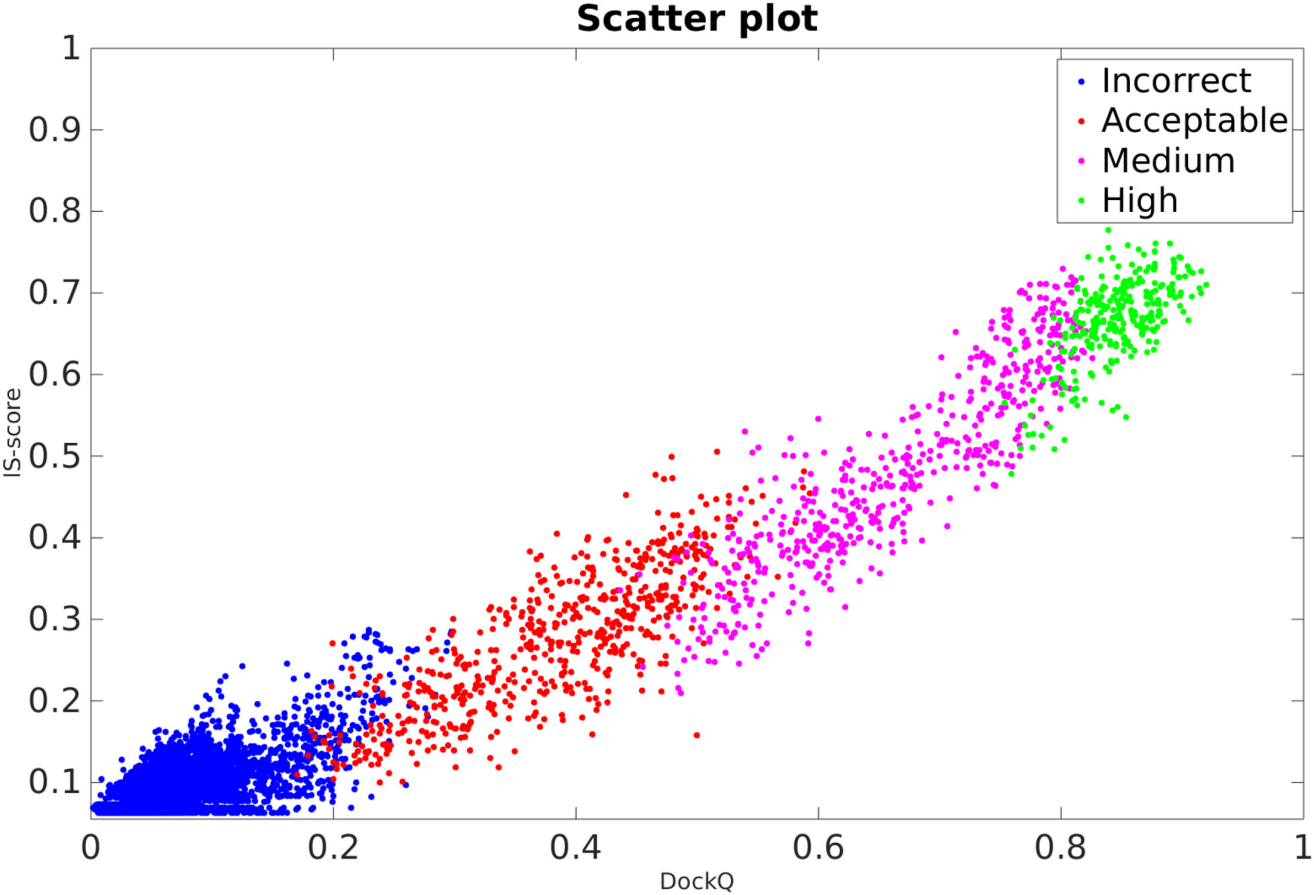
Scatter plot IS-score vs. DockQ on the CAPRI-set. Models are colored according to CAPRI classification as Incorrect (blue), Acceptable (cyan), Medium (red), High (green). The overall correlation is R = 0.98, while the correlation within the different quality classes is 0.77, 0.82, 0.90, and 0.65, respectively.

**Fig 3:**
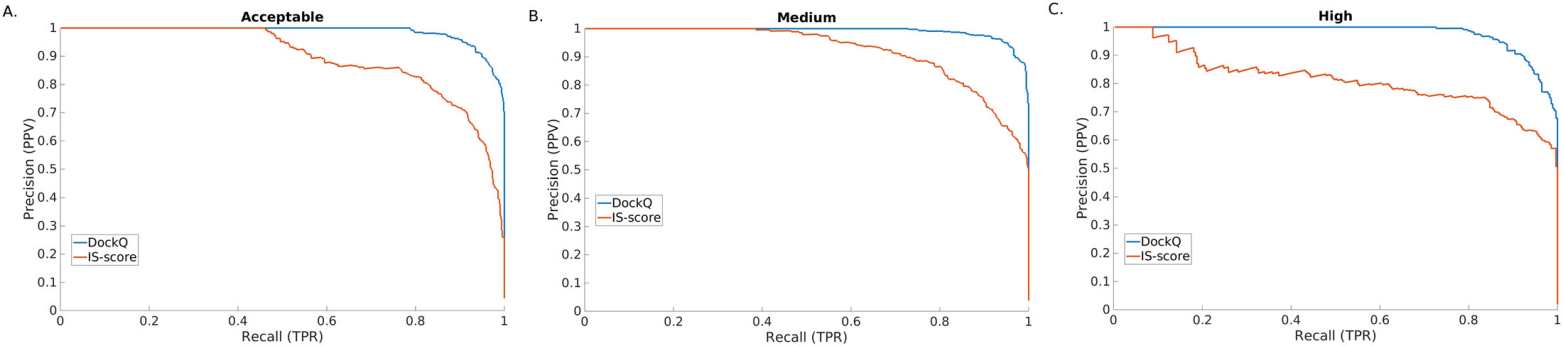
Precision (PPV) vs. Recall plots for the ability of DockQ and IS-score to separate models with (A) Acceptable or better, (B) Medium or better, and (C) High quality, respectively, on the CAPRI-set. The area under the curves (AUC) for DockQ and IS-score are (0.98, 0.99, 0.97) and (0.89, 0.92, 0.82) respectively for (A) Acceptable, (B) Medium and (C) High.

The CAPRI-set was used as an independent benchmark to assess DockQ performance and compare it to IS-score, which is similar in its design to TM-score for protein structure prediction, but for interfaces. The cutoffs optimized on MOAL-set are close to optimal also for the CAPRI-set within ±0.02 (Fig 1B), showing that cutoffs optimized on the MOAL-set can also be used on the CAPRI-set. However, the main purpose of giving the cutoffs is to show the general correspondence between DockQ and CAPRI classification not to use the cutoffs for classification. Even though DockQ and IS-score has an overall Pearson's correlation of 0.98 (Fig 2), the ability to reproduce the CAPRI classification is much better for DockQ. As illustrated in Fig 2, the separation between different quality classes is much better according to DockQ, while the separation based on IS-score is much more overlapping, e.g. an IS-score of 0.5 actually have models in three different classes, Acceptable, Medium and High. Precision (PPV) vs. recall (TPR) curves were then constructed to compare in greater detail, the ability of the two methods to classify the models with respect to their original CAPRI classification: Acceptable or better, Medium or better and High, by varying the cutoffs in the whole range [0, 1] of DockQ and IS-score (Fig 3). The area under the curves (AUC) for DockQ (0.98, 0.99, 0.97) show almost perfect agreement with respect to the original CAPRI classification. This is true across all quality classes; e.g. the PPV for DockQ at a recall of 90% is 95%, 97%, and 91% for Acceptable, Medium and High respectively, while the PPV for IS-score is 71%, 72%, and 66% at the same recall. It is no surprise that agreement with CAPRI classification is exceptionally good for DockQ since it is using the CAPRI measures to derive the score. In fact, the average DockQ for false predictions are within ±0.02 of the cutoff for a particular class, which means that most false predictions are borderline cases. This is of course a consequence of classifying models in different quality bins, for instance taking the original CAPRI classification as golden standard, the average iRMS for the models with Medium quality classified incorrectly as High by DockQ is 1.05Å, and High quality classified incorrectly as Medium is 0.93Å, while the cutoff in iRMS between medium and high quality is 1.0Å (Medium < 1.0Å; High ≥ 1.0Å) according to CAPRI [10]. In any classification scheme there will be borderline cases, where virtually identical models are classified differently. Highlighting, yet again, the importance of using continuous measures like DockQ or IS-score, which do not exhibit the same problems.

### Software feature to deal with interacting multi-chains

Assessing dimer quality is a current challenge in CASP and CAPRI. In view with this, the DockQ software has been built with the functionality to deal with interacting multi-chains. Monomer-dimer or dimer-dimer interfaces are common in, for example, antigen-antibody interactions, due to the internal symmetry in the biological assembly of the heavy and light variable chains of the immunoglobulin, where the partner-antigen can potentially bind asymmetrically at the antigen binding sites [20]. This is also common amongst molecular recognition involved in Major Histocompatibility Complexes in antigen presenting cells [21], nuclear transport and other signal transduction pathways [22]. Multimeric biological assemblies of higher order than that of dimers are also found to occur, particularly common in viral envelopes / capsids [23], viral glycoproteins [24,25] and cytoplasmic subunits of voltage-gated channels [26]. To this end, the software has been built with the functionality to handle all different possible combinations of chains specified in the two inputs (native, model) with appropriate command-line options without the need to merge the chains manually before. It also has the option to tryout different chain order combinations to find the best matching DockQ score if there are multiple symmetric correct solutions.

## Conclusions

DockQ is a continuous protein-protein docking model quality score, performing as good as the three original CAPRI measures (F_nat_, LRMS, iRMS) in segregating the models in the four different CAPRI quality classes. If the CAPRI measures are already calculated it is simple to calculate DockQ using Eq 2 with d_1_ = 8.5 and d_2_ = 1.5. Since DockQ essentially recapitulates the CAPRI classification almost perfectly, it can be viewed as a higher resolution version of the CAPRI classification. The fact that it is continuous makes it possible to estimate model quality in a more quantitative way using Z-scores or sum of top ranked models, which has been so valuable for the CASP community. It should also be very useful for comparing the performance of energy functions used for ranking and scoring docking models in more detail, by analyzing complete rankings (not only top ranked), correlations, and DockQ vs. energy scatter plots. In addition, DockQ can be used as a target function in developing new knowledge-based scoring functions using for instance machine learning, a feature that has been investigated in a separate study [16]. To simplify the calculation of DockQ we provide a stand-alone program that given the atomic coordinates of a docking model and the native structure calculates all CAPRI measures and the DockQ score.

## Supporting Information

S1 File Supporting Figures. **Figure A.** Demonstration of the Inverse Square Scaling technique. The scaling parameter, k, describes the raw score (X) at half maximal high (0.5) of the scaled score, Y. k is set to 8.5 in this example illustration which is the optimized value for LRMS. **Figure B.** Heat map with average F1-values for the optimization of d_1_ and d_2_ on the MOAL-set. Each value is smoothed by taking an average over its nearest neighbors to remove the effect of outliers.
(PDF)
