# Supplementary material for: DockQ: A Quality Measure for Protein-Protein Docking Models
Source: PLoS One. 2016 Aug 25;11(8):e0161879. doi: 10.1371/journal.pone.0161879 (PMC4999177; doi:10.1371/journal.pone.0161879)
Supplement: S1 File — Figure A. Demonstration of the Inverse Square Scaling technique. The scaling parameter, k, describes the raw score (X) at half maximal high (0.5) of the scaled score, Y. k is set to 8.5 in this example illustration which is the optimized value for LRMS. Figure B. Heat map with average F1-values for the optimization of d1 and d2 on the MOAL-set. Each value is smoothed by taking an average over its nearest neighbors to remove the effect of outliers. (PDF) [file pone.0161879.s001.pdf]

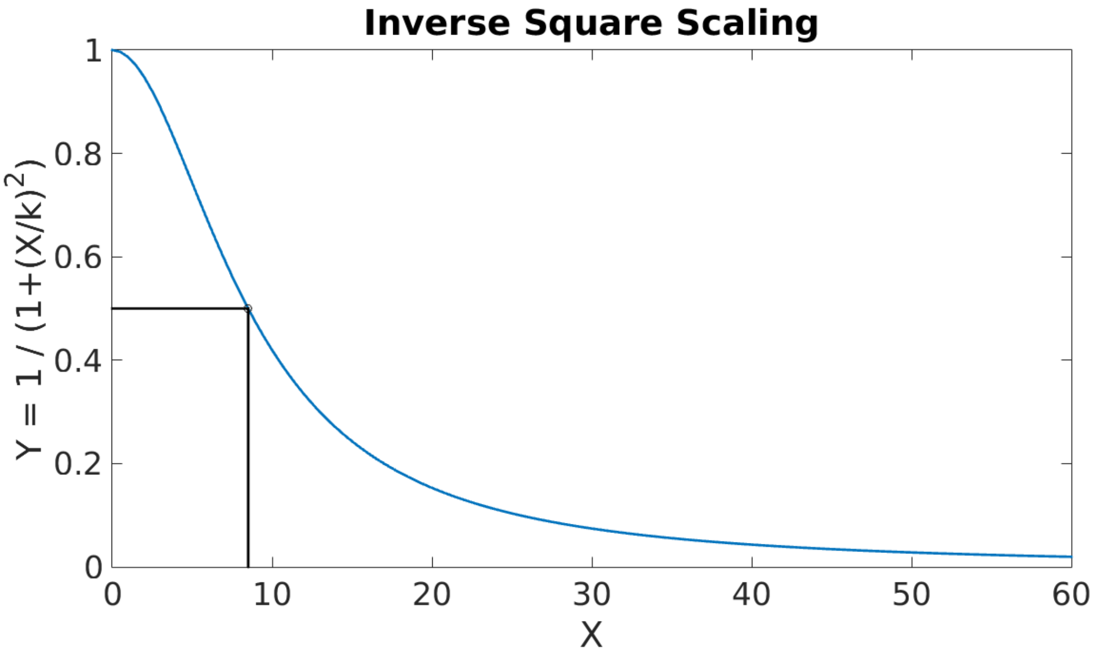

Figure A. Demonstration of the Inverse Square Scaling technique. The scaling parameter, k, describes the raw score (X) at half maximal hight (0.5) of the scaled score, Y. k is set to 8.5 in this example illustration which is the optimized value for LRMS.

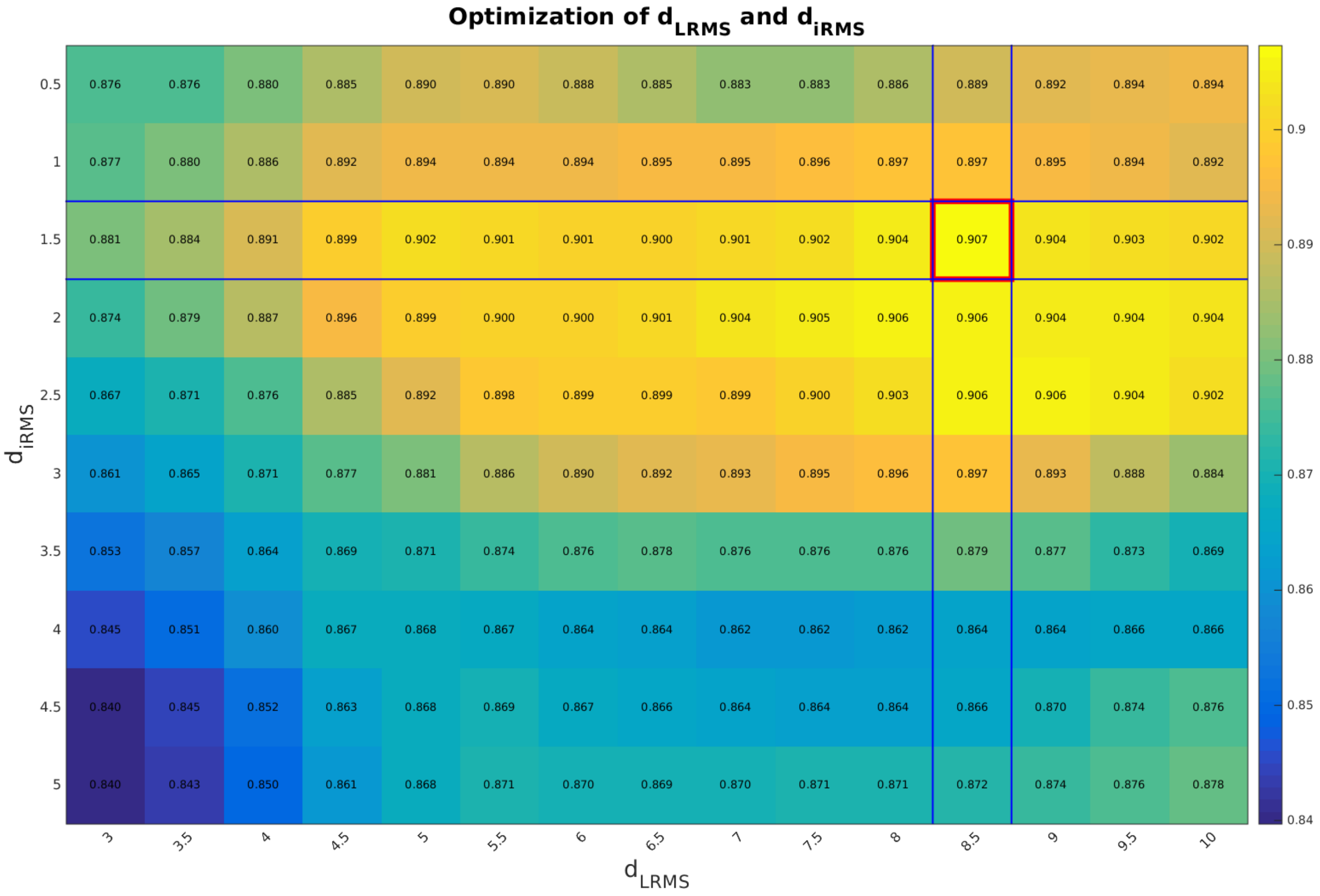

Figure B. Heat map with average F1-values for the optimization of d_1 and d_2 on the MOAL-set. Each value is smoothed by taking an average over its nearest neighbors to remove the effect of outliers.
